# Reprogramming of the estrogen responsive transcriptome contributes to tamoxifen-dependent protection against tumorigenesis in the p53 null mammary epithelial cells

**DOI:** 10.1371/journal.pone.0194913

**Published:** 2018-03-28

**Authors:** Murugesan Palaniappan, David Edwards, Chad J. Creighton, Daniel Medina, Orla M. Conneely

**Affiliations:** 1 Department of Molecular and Cellular Biology, Baylor College of Medicine, Houston, TX, United States of America; 2 Division of Biostatistics, Dan L. Duncan Cancer Center, Baylor College of Medicine, Houston, TX, United States of America; Roswell Park Cancer Institute, UNITED STATES

## Abstract

The tumor suppressor gene p53 is frequently mutated in human breast cancer and is a marker for poor prognosis and resistance to chemotherapy. Transplantation of p53 null mouse mammary epithelium into syngeneic wild-type mice leads to normal mammary gland development followed by spontaneous mammary tumors that recapitulate many of the phenotypic, molecular and genetic features of human breast cancer. Transient exposure of p53 null mice to the anti-estrogen, tamoxifen leads to sustained and robust protection against tumor development. However the mechanism underlying this anti-tumor activity remains poorly understood. Here we demonstrate that transient exposure to tamoxifen leads to a reduction in mammary ductal side-branching and epithelial cell proliferation after tamoxifen withdrawal. Global gene expression analysis showed that transient tamoxifen exposure leads to persistent changes in the expression of a subset of estrogen regulated gene signatures in mammary epithelial cells (MECs). Among these was the protein tyrosine phosphatase, non-receptor type 5 (*Ptpn5*). We show that *Ptpn5* is a novel tamoxifen regulated target gene which is upregulated in MECs after transient tamoxifen exposure and displays tumor suppressor activity in human breast cancer cells. Further, PTPN5 expression is strongly associated with good clinical outcome in tamoxifen treated human breast cancer patients suggesting that PTPN5 may represent a novel biomarker of tamoxifen response in human breast cancer.

## Introduction

Breast cancer (BC) comprises a heterogeneous group of diseases that can be discriminated at the molecular level into approximately six distinguishable subtypes based on genome-wide transcription profiling [1–3]. Estrogen receptor α (ERα) and progesterone receptors (PRs) play a central role in regulating both postnatal development of the mammary gland and breast cancer by promoting proliferation of mammary epithelial and breast cancer stem/progenitor cells [4–7]. The majority (~75%) of breast cancers are ERα positive, hormone-dependent for growth and responsive to endocrine therapy [8]. Endocrine therapy using aromatase inhibitors (AIs) to block estrogen production or antiestrogen selective estrogen receptor modulators (SERMS) remain the most widely used and most effective treatment for breast cancers in women who have tumors positive for ERα [9]. Further, both SERMS and AIs have been shown to confer substantial protection (>50%) against ER+ breast cancer development leading to FDA approval of tamoxifen and raloxifene to reduce BC risk in women at high risk of BC development [10–13].

The tumor suppressor gene p53 is one of the most frequently mutated genes in breast cancers occurring at a frequency of >25% in luminal and >80% in triple negative BCs [3] and *p53* mutations have been associated with poor prognosis and resistance to chemotherapy [14, 15]. While the vast majority (~75%) of p53 mutations are associated with loss of p53 function, a subset of mutations also lead to aberrant gain of oncogenic functions [16–19]. The loss or mutation of p53 in mice leads to enhanced tumorigenic potential by promoting cell proliferation and genomic instability [20–23]. p53 null mice die at 4 to 5 months of age due to lymphosarcomas but do not develop mammary tumors at an appreciable level [22, 23]. To directly address the consequences of p53 loss on mammary tumorigenesis, a p53 null mammary epithelium transplantation model has been developed. Transplantation of p53 null mammary epithelium into cleared fat pads of wild-type (WT) mice leads to normal mammary gland development followed by development of spontaneous mammary tumors that recapitulate many of the phenotypic, molecular and genetic features of human breast cancer [24–26]. The normal p53 null mammary epithelium is ERα positive and responsive to ovarian steroid hormones that contribute to growth, functional differentiation and tumorigenesis [20]. Furthermore, gene expression profiling for classification indicates that, like human tumors, p53 null mouse mammary tumors fall into multiple molecular groups, including luminal, basal-like and claudin-low subtypes [25–27]. Blockade of estrogen signaling by ovariectomy or antiestrogen treatment strongly reduces mammary tumorigenesis in p53 null mammary epithelium indicating that ovarian steroid hormones are required for tumorigenesis in p53 null mammary cells [28]. Indeed, short term exposure to tamoxifen in the p53 null model confers robust (>90%) and sustained protection against mammary tumorigenesis [28]. However, the cellular and molecular events associated with tamoxifen-induced protection against mammary tumorigenesis in the p53 null model remain poorly understood.

In the present study, we used genome-wide transcription profiling to determine whether persistent global changes in the ERα responsive transcriptome contribute to the tumor protective effects observed after transient exposure of p53 null mammary glands to tamoxifen. We disclose the intrinsic changes in the ERα responsive genes that are associated with tamoxifen-dependent tumor protection and we address the functional contribution of a novel tamoxifen regulated target gene, the protein tyrosine phosphatase, PTPN5 to tumor suppression in a xenograft model of p53 mutant human BC.

## Materials and methods

### Mice

All donor and recipient mice were bred and maintained at Baylor College of Medicine. The donor mice were Balb/c p53-null, and the recipient mice were Balb/c p53 WT. All mice were maintained in a conventional mouse facility with room temperature set at 22°C with food and water provided *ad libitum*. The animal facility is accredited by the American Association of Laboratory Animal Care and all the animal experiments were approved by the Institutional Animal Care and Use Committee (IACUC) at Baylor College of Medicine.

#### Transplantation

The basic transplantation protocol was previously described by Jerry and colleagues [24]. Briefly, the inguinal (#4) mammary fat pads of three week old Balb/c WT mice were cleared. Five weeks after fat pad clearance, 1-mm^2^ fragments of mammary duct from 8 week old female p53-null mice were transplanted into both cleared inguinal mammary fat pads. The transplanted cells take 8 weeks to completely fill the fat pad, at which point the cells assume a steady-state level of proliferation. Thus, tamoxifen treatment was started at 16 week old hosts’ age to avoid any effects of the agents on the ability of the cells to grow and fill the fat pad. At 16 weeks of host age, mice received a 5mg tamoxifen (TAM) pellet or sham (control) pellet (Innovative Research of America, Sarasota, FL) subcutaneously (SC) on the back. The pellets remained in place for 13 weeks before removal. At 4 weeks or 8 weeks after cessation of tamoxifen, all mice were injected with 17β-Estradiol (100μg SC). After 8h of 17β-Estradiol (E_2_) treatment, all mice were sacrificed and #4 mammary gland transplants were collected. Schematic diagram of the p53 null transplantation was shown in S1A Fig. Mammary epithelial cells (MECs) were isolated by collagenase digestion as described previously [26].

#### Total RNA isolation

For microarray and quantitative Real-Time PCR (qPCR), total MEC RNA or total mammary gland RNA was isolated from #4 glands (lymph nodes removed) using the RNeasy Lipid Tissue Midi Kit according to the manufacturer’s instructions (QIAGEN, Inc., Valencia, CA).

#### Affymetrix microarray analysis

Microarray analysis was performed on MEC total RNA obtained from sham and tamoxifen treated groups by using Affymetrix Gene Chip Mouse Gene 1.0 ST array (Affymetrix Inc. Santa Clara, CA). Microarray was carried out as previously described [29]. To ensure statistical significance, samples from sham and tamoxifen treated groups were tested in triplicate. For the microarray data analysis, the estimated False Discovery Rate (FDR) for the top set of array probes with nominal p<0.01 and fold change>1.4 was <5% (By using the method of Storey and Tibshirani [30] on the full set of probes with fold change>1.4). The microarray raw data are deposited into GEO (accession number GSE77948).

#### Quantitative Real-Time PCR

The microarray results were validated by qPCR and performed as previously described [29]. TaqMan Primer probes were purchased from Applied Biosystems (Foster City, CA) and are as follows: *Ptpn5 (Mm00479063_m1)*, *Accn1 (Mm00475691_m1)*, *Fgf12 (Mm00679872_m1)*, *Krt4 (Mm01296260_m1)*, *Areg (Mm00437583_m1)*, *Nrxn3 (Mm04279482_m1) and Ppid (Mm00835365_g1)*. All experiments were performed in triplicate using three independent cDNA sets per treatment and normalized to cyclophilin D (*Ppid*).

#### Western blot analysis

Western blot analyses were performed as previously described [31]. Primary antibodies included PCNA (Cell Signaling #2586, 1:1000), Ki67 (Santa Cruz, sc-7846, 1:1000), cyclin D1 (Thermo Scientific, RB-212-P, 1:2000), actin (Santa Cruz, sc-1615, 1:5000), PTPN5 (Thermo Scientific, PA5-15531, 1:2000), Phospho-p44/42 MAPK (Erk1/2) (Thr202/Tyr204) (Cell Signaling #9101, 1:2000) and total MAPK (Cell Signaling #4695, 1:2000).

#### Mammary gland whole mount

Whole-mount staining of mammary tissue was performed as previously described [32].

#### Immunohistochemistry analysis

Immunohistochemistry analysis and quantitation were performed as previously described [29]. Primary antibodies included Ki67 (Abcam, ab15580, 1:5000), cyclin D1 (Thermo Scientific, RB-9041-P, 1:200), progesterone receptor (PR) (A0098, 1:50; DAKO) and ERα (Santa Cruz, sc-542, 1:50). The secondary antibody was goat anti-mouse biotinylated (Jackson ImmunoResearch, 115-065-146, 1:400).

#### Validation of Ptpn5 ERα binding sites by ChIP-qPCR

WT mice (10 weeks old) were ovariectomized and rested for 10 days. Mice were injected SC with sesame oil (50μl) or E_2_ (100ng) for 8h and then mice were sacrificed and both #4 mammary glands (lymph nodes removed) were harvested from each mouse. Mammary gland chromatin was prepared using ChIP-IT High Sensitivity Kit (Active Motif, Carlsbad, CA) according to the manufacturer’s instructions. ERα ChIP enrichment was verified by qPCR using SYBR Green Master Mix. *Ptpn5* primer corresponding to the region identified by ERα ChIP-seq was generated. Primer sequences are as follows: *mPtpn5* +24670 Forward- GGCCGTGTCATCCCACTCT and *mPtpn5* +24670 Reverse GCCAGGCTGGGTGACTCA. The enrichment of ERα binding found in each sample was normalized to input values.

### Cell culture

MCF7 parental cells were cultured in RPMI supplemented with 10% of FCS and 1% penicillin/streptomycin. The MCF7 tamoxifen resistance cell line was established from a long-time treatment of 100nM 4-hydroxytamoxifen (4-OHT) (Sigma) until cell growth resumed. Tamoxifen resistant cells were cultured in phenol red-free medium containing 10% charcoal-dextran-stripped FCS, 100nM 4-OHT and 1% penicillin/streptomycin. The tamoxifen resistance cell line was authenticated once resistance was established, and mycoplasma contamination testing was performed once every 6 months. MDA-MB-231 human breast cancer cells were cultured in DMEM supplemented with 10% FBS and 1% penicillin/streptomycin. MDA-MB-231 stable cell line expressing PTPN5 cDNAs were generated by lentiviral transduction in the presence of 8 μg/mL polybrene followed by selection with appropriate antibiotic resistance markers. All cell lines were incubated at 37°C and 5% CO_2_.

### Plasmid construction

For transient overexpression experiments, the PTPN5-coding sequence was PCR amplified with the addition of restriction sites Xmal and Not1 and cloned into pcDNA3.1 vector (Invitrogen, Grand Island, NY, USA). The PTPN5 construct was sequence verified prior to transient overexpression experiments. For stable expression of PTPN5 experiments, pInducer 21 lentiviral vector was kindly provided by Dr. Thomas Westbrook [33]. The PTPN5-coding sequence was PCR amplified and cloned with Gateway cloning as described previously [33]. All plasmids were sequence-verified prior to lentiviral production. For lentiviral production and titering, pInducer 21 lentiviral vector was co-transfected with packaging vectors into 293-T cells using Fugene 6. Viral supernatant was collected at 48 and 72 h post-transfection, pooled and filtered through 0.45 μM filters to remove cellular debris. Filtered viral supernatant was concentrated using the Beckman Coulter Optima ultracentrifuge (SW32Ti rotor) at 25,000 rpm for 1h and 45 min. Ultracentrifuged virus was resuspended in DMEM. MDA-MB-231 cells were transduced with lentivirus and selected with neomycin. After neomycin selection, cells were aliquoted and frozen for experiments.

### Cell growth assay

MDA-MB-231 were engineered with doxycycline (Dox)-inducible PTPN5 cDNA and plated at 10,000 cells/well in 12-well plates. Cells were treated with or without Dox for 0, 3, 5 and 7 days. The medium and Dox were replenished every 2 days. Cells were washed with Hanks' buffered salt solution (Invitrogen), lifted from plates with 0.25% EDTA Trypsin (Invitrogen), placed in Isoton II Diluent (Beckman) with 3 drops of Zapoglobin II Lytic reagent (Beckman), and counted using a Coulter particle counter Z1 (Beckman).

### *In vivo* MDA-MB-231 breast cancer xenograft study

Orthotopic tumorigenicity assays were performed as previously described [34]. Briefly, MDA-MB-231 cells (containing inducible stable expression of PTPN5) were harvested by trypsinization, washed twice in PBS and counted. Cells were then resuspended in PBS. Athymic Nude mice (6 weeks old) were anesthetized, a small incision was made to reveal the mammary gland and 1X10^6^ cells were injected directly into the mammary fat pad (#4 mammary gland right side only). The incision was closed with wound clips and mice received drinking water with or without Dox. Primary tumor outgrowth was monitored weekly by taking measurements of the tumor length (*L*) and width (*W*).

### Bioinformatics

Enriched gene ontology (GO) terms were identified using the DAVID Functional Annotation Tool (http://david.abcc.ncifcrf.gov/summary.jsp) [35]. The online database was employed to determine the relevance of PTPN5 mRNA expression to the overall survival [36].

### Statistical analysis

Data are presented as mean±SEM. The significance of the differences between groups was determined by Student's *t*-test and one-way ANOVA followed by the Tukey multiple comparison tests using Prism software (GraphPad Prism, version 5, GraphPad Inc., San Diego, CA). Values were considered statistically significant at *P* < 0.05.

## Results

### Transient exposure to tamoxifen reduces mammary ductal side-branching and decreases MEC proliferation

**Fig 1:**
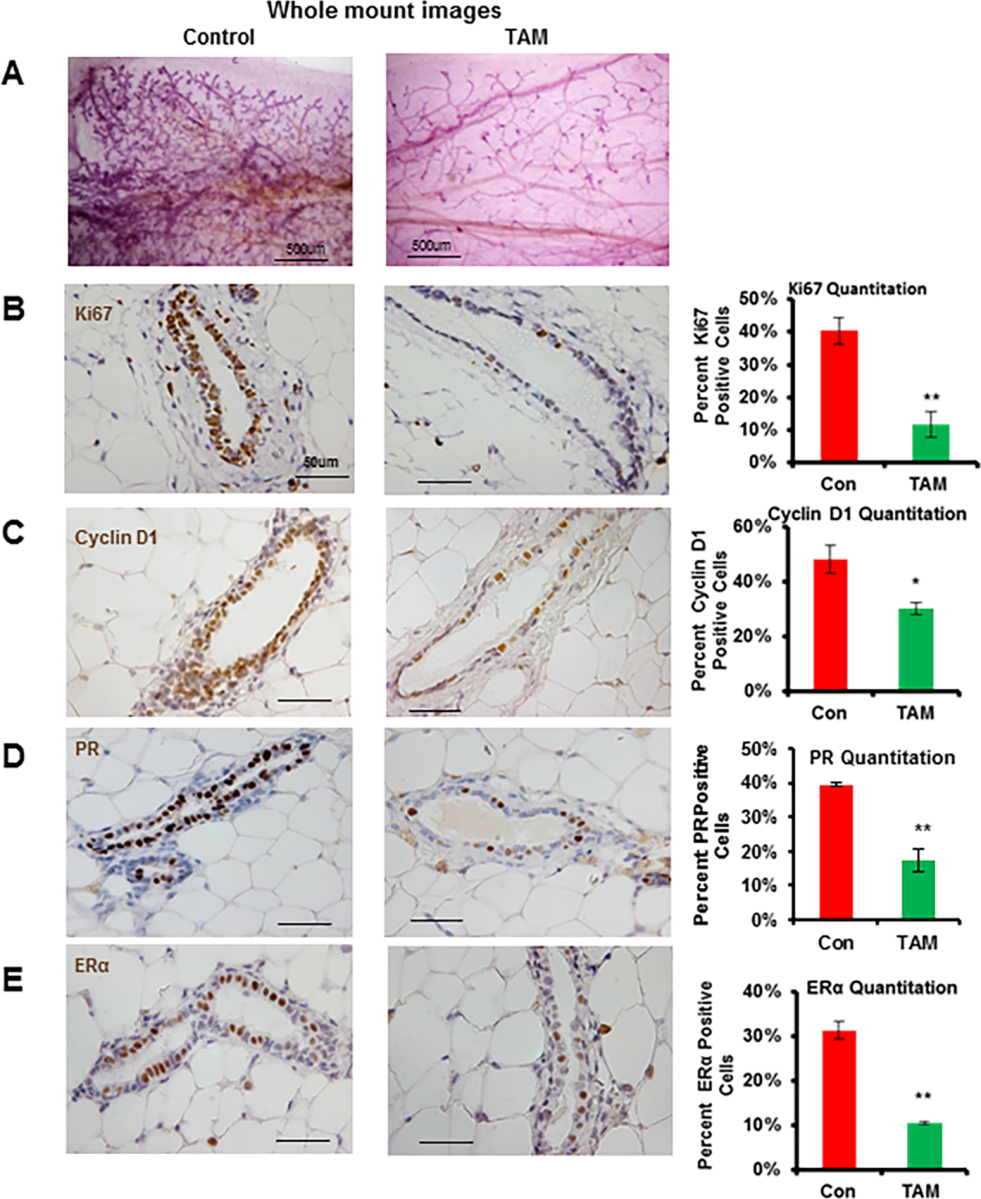
Transient exposure to tamoxifen inhibits mammary ductal side-branching, proliferation, PR and ERα in the p53 null mammary gland. (A) Mammary epithelium from p53 null mice was transplanted into the cleared fat pad of syngeneic WT Balb/c mice. At 8 weeks after transplantation, mice received a tamoxifen (TAM) (5mg) or sham (control) pellet (SC) for 13 weeks. Four weeks after tamoxifen removal, all mice were treated with E_2_ (100ug) for 8 h and whole-mount images of carmine-stained p53 null mammary glands. Scale bars correspond to 500μm. A total of 6 mice per treatment group were used. (B-E) Representative immunohistochemical staining for Ki67, cyclin D1, PR and ERα on paraffin-embedded p53 null transplanted mammary gland sections from sham and tamoxifen treated mice. Scale bars correspond to 50 μm. A total of 5 mice were used per treatment group. Graphs representing the ratio of Ki67, cyclin D1, PR and ERα positive cells in p53 null mammary epithelium. Data are mean ± SEM of 5 mice. *, *P* < 0.05; **, *P* < 0.01.

To examine the consequences of transient exposure to tamoxifen on p53 null mammary gland development, we transplanted p53 null mammary epithelium from 8 week old p53 null Balb/c mice into the cleared fat pad of syngeneic WT Balb/c mice. Eight weeks after transplantation, mice received a tamoxifen pellet (5mg) or sham pellet subcutaneously (SC) that was removed after 13 weeks. Four weeks after tamoxifen withdrawal, transplanted p53 null mammary glands were collected for whole-mount analysis. At this stage, tamoxifen has been cleared from the system and we confirmed normal estrus cycling of mice by vaginal smears. As shown in Fig 1A and S1B Fig, sham treated mice contained fad pads completely filled by the mammary tree with extensive side-branching. However, in the mammary gland of tamoxifen treated mice, the mammary tree still filled the entire fat pad but dramatically reduced side-branching was observed, indicating that transient exposure to tamoxifen leads to a persistent reduction in mammary ductal side-branching in the p53 null mammary gland.

To address the cellular mechanisms associated with the observed decrease in mammary ductal side-branching, we asked whether transient exposure to tamoxifen reduced MEC proliferation. To test this, we examined the pattern of Ki67 and cyclin D1 expression in transplanted p53 null mammary glands by immunohistochemical staining. The ductal epithelium of mice exposed to transient tamoxifen treatment displayed a significant reduction in the frequency of Ki67 and cyclin D1 positive MECs relative to sham exposed mice (Fig 1B and 1C). Furthermore, Western blot analysis of the expression of proliferative markers such as PCNA, Ki67 and cyclin D1 in MECs obtained at 8 weeks after tamoxifen withdrawal revealed that the decrease in proliferation was sustained long term (S2 Fig), indicating that transient exposure to tamoxifen leads to a persistent reduction in MEC proliferation after withdrawal of tamoxifen. The ovarian hormones, estrogen and progesterone act as master regulators of mammary gland development. Specifically, estrogen triggers ductal elongation during puberty whereas progesterone involved in mammary gland side-branching [37–39]. Analysis of the consequences of tamoxifen exposure on PR and ERα expression by immunohistochemistry showed that the frequency of MECs expressing both receptors decreased after tamoxifen exposure (Fig 1D and 1E) and may contribute to the suppression of mammary ductal side-branching and proliferation.

### Transient exposure to tamoxifen leads to changes in estrogen responsive gene signatures in MECs

**Fig 2:**
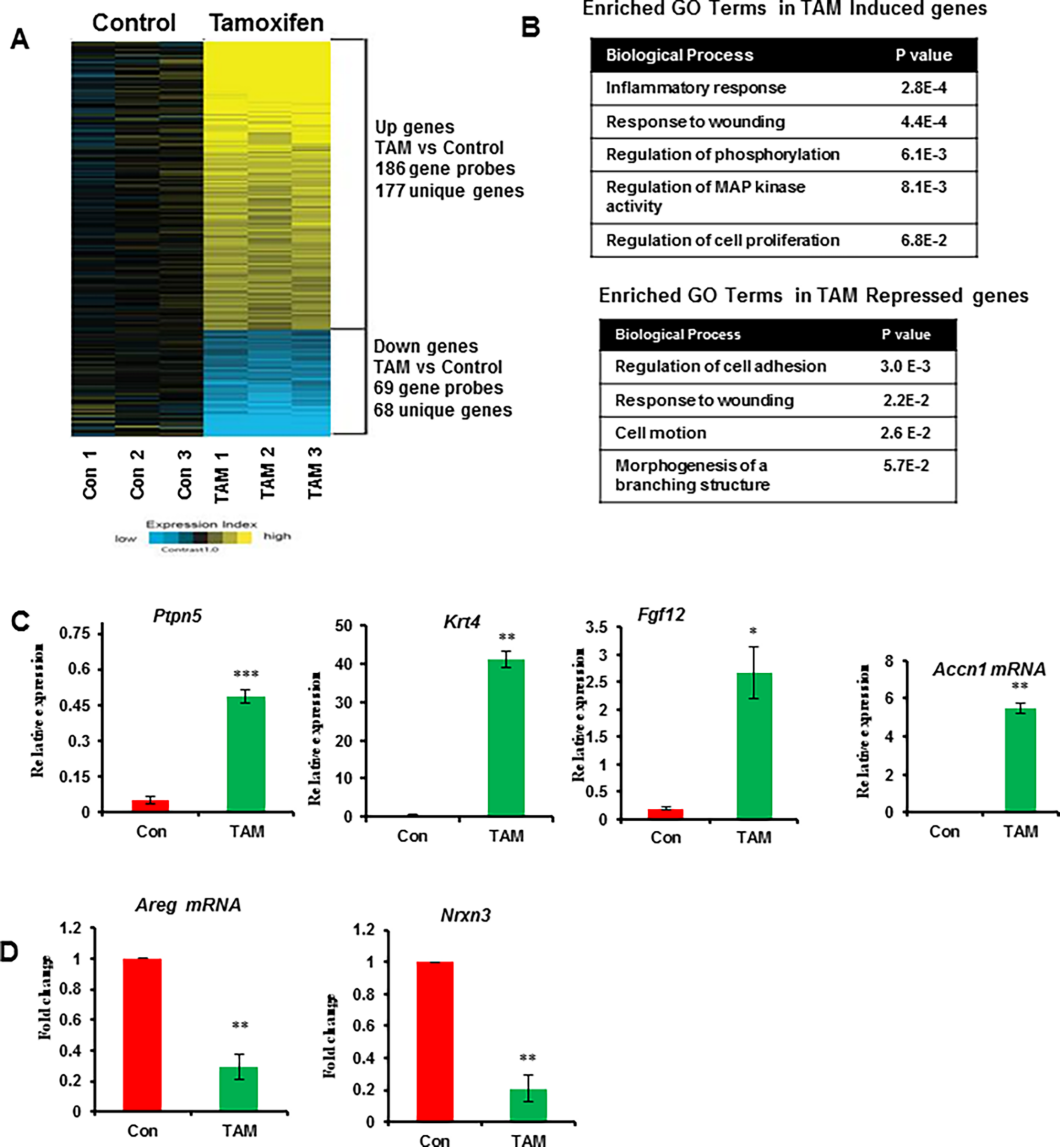
Transient exposure to tamoxifen alters ERα responsive gene expression signatures in p53 null MECs. At 4 weeks after tamoxifen removal, all mice were treated with E_2_ (100ug) for 8 h and MECs were isolated for global gene expression analysis. (A) Heatmap of 245 genes (*P* < 0.01 and FC > 1.4) that are differentially expressed between p53 null MECs samples from transient exposure to tamoxifen treatment. Triplicate pools of p53 null MECs (5 mice per pool) were used for array analyses under each treatment condition. For the heat map, log2 expression values were centered on the average of the control group. “Expression index” represents log2 fold change from control group, where bright yellow or blue represents >2-fold change or <0.5-fold change, respectively, from control. (B) Summary of enriched GO terms of tamoxifen induced and repressed genes using the DAVID Functional Annotation Tool. For DAVID functional annotation, we used p<0.01 and fold change>1.4. The complete gene sets (up and down genes) were shown in S1 Table. (C and D) Quantitative Real-Time PCR validation of tamoxifen induced and repressed genes in p53 null MECs. Results are means ± SEM of three independent experimental replicates. *, *P* < 0.05; **, *P* < 0.01; ***, *P* < 0.001.

To determine whether the morphological changes observed in the mammary epithelium after tamoxifen exposure are reflected in changes in intrinsic estrogen responsive gene signatures in p53 null MECs, we next performed global gene expression analysis by microarray profiling of MECs isolated from control and tamoxifen exposed mice 4 weeks after tamoxifen withdrawal and treated with E_2_ for 8h. We identified 245 differentially regulated genes (*P*<0.01 and FC>1.4). Of these, 177 genes (72%) were persistently upregulated and 68 genes (28%) were persistently downregulated after transient exposure to tamoxifen (Fig 2A and S1 Table). To identify the biological function of the differentially regulated genes, we used DAVID functional annotation analysis. Interrogation of the 177 genes induced by transient exposure to tamoxifen identified a significant enrichment for GO terms associated with inflammatory response, response to wounding, regulation of phosphorylation, regulation of MAP kinase activity and regulation of cell proliferation. Genes repressed by tamoxifen were enriched for GO terms associated with regulation of cell adhesion, response to wounding, cell motion and morphogenesis of a branching structure (Fig 2B). Finally, we independently validated differential expression of several upregulated and downregulated genes by qPCR (Fig 2C and 2D). Furthermore, qPCR analysis of tamoxifen-induced genes *(Ptpn5*, *Accn1*, *Krt4 and Fgf12*) in p53 null MECs obtained 8 weeks after tamoxifen withdrawal revealed that the increased mRNA levels were sustained over the long term (S3 Fig). These results indicate that transient exposure to tamoxifen leads to enduring intrinsic changes in gene expression profiles of p53 null MECs that persist after tamoxifen withdrawal.

### Integration of tamoxifen regulated gene signatures with molecular profiles that distinguish outcomes in response to tamoxifen therapy in human BC patients identifies PTPN5 as a potential novel biomarker of tamoxifen response

**Fig 3:**
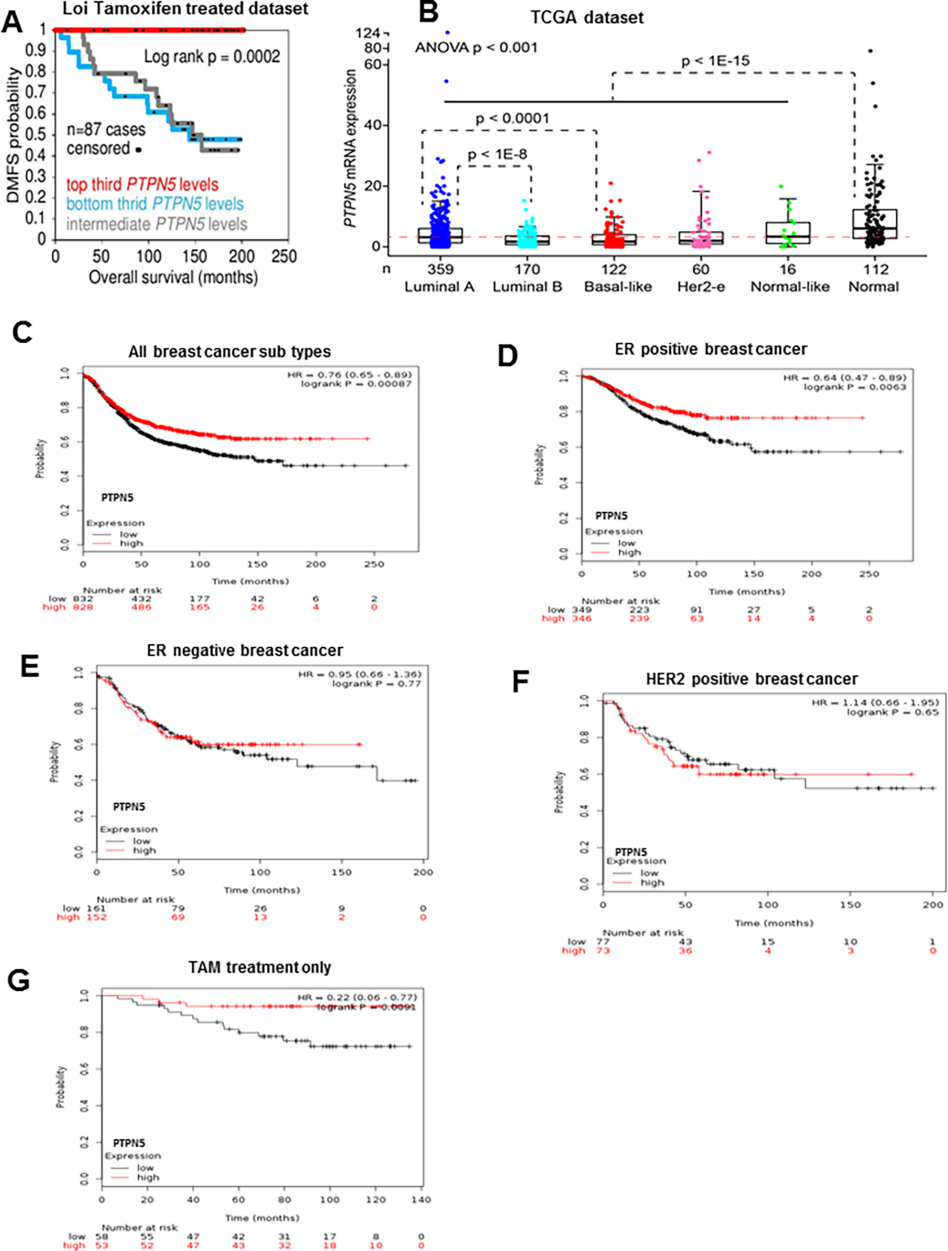
PTPN5 expression is associated with good clinical outcome in breast cancer patients. (A) Kaplan-Meier survival curve of 87 breast cancer patients who received adjuvant tamoxifen treatment. The x axis indicates time in months and y axis shows distant metastasis-free survival. (B) Boxplot showing the distribution of PTPN5 expression in various subtypes of breast cancer patients and the normal from healthy women within the TCGA cohort. The x axis indicates number of individual utilized and y axis shows PTPN5 mRNA levels. (C) Survival curves are plotted in all breast cancer patients (n = 1660). (D) Survival curves are plotted for ER positive breast cancer patients (n = 695). (E) Survival curves are plotted for ER negative cancer patients (n = 313). (F) Survival curves are plotted in HER2 breast cancer patients (n = 150). (G) Survival curves are plotted in tamoxifen treated patients (n = 111).

In order to identify novel biomarkers of tumor protection, we integrated the tamoxifen regulated gene signatures with gene expression signatures obtained from 255 ER+PR+ breast cancer patients who had received tamoxifen monotherapy for 5 years and were followed for response using distant metastasis free survival (DMFS) as an endpoint [40]. We identified a subset of our tamoxifen upregulated genes were associated with good prognosis in these patient molecular profiles (S2 Table). Furthermore, we discovered that Ptpn5 is the top most genes in this analysis. In addition, we independently confirmed that *Ptpn5* is persistently upregulated in p53 null MECs after transient exposure to tamoxifen (Fig 2C, S3 Fig and S4 Fig). Therefore, we focused our attention on *Ptpn5*, a member of the non-receptor tyrosine phosphatase family of enzymes that has not previously been functionally characterized in BC. Although one member of this family, PTPN12, has been characterized as a negative regulator of growth factor/receptor tyrosine kinase signaling in human BC cells and has been implicated as a tumor suppressors of triple negative BC [41]. PTPN5 has not previously been functionally characterized in BC. We independently confirmed that *Ptpn5* is persistently upregulated in p53 null MECs after transient exposure to tamoxifen (Fig 2C, S2 Fig and S4 Fig). As shown in Fig 3A, high levels of PTPN5 were strongly correlated with DMFS and overall survival after adjuvant tamoxifen treatment.

**Fig 4:**
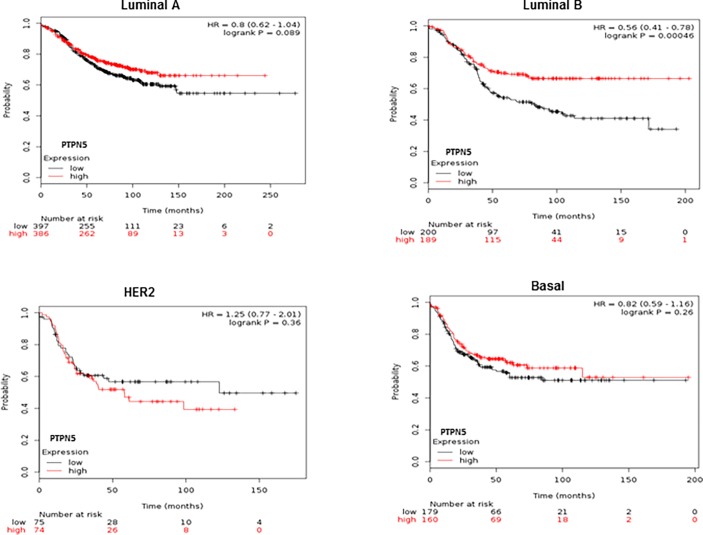
Prognostic value of PTPN5 mRNA expression in different subtype of breast cancer patients. Survival curves are plotted in luminal A (n = 783), luminan B (n = 389), HER2 (n = 149) and basal (n = 339) subtypes breast cancer patients by using the Kaplan-Meier plotter database (http://kmplot.com/analysis/index.php?p=service&cancer=breast).

To further explore the clinical significance of PTPN5 expression in human breast cancer patients, we queried the TCGA dataset to examine levels of PTPN5 expression in distinct molecular subtypes of BC [3]. As shown in Fig 3B, *PTPN5* mRNA expression was significantly reduced in all subtypes of breast cancer patients when compared to normal samples. In addition, we also assessed the prognostic value of PTPN5 mRNA expression in various breast cancer subtypes using the Kaplan Meier Plotter database. High levels of PTPN5 expression were strongly associated with good overall survival for all breast cancer patients which were followed for 20 years (Fig 3C, p<0.0008). Further, within the 695 ER positive breast cancer cases, high levels of PTPN5 expression were strongly correlated with good overall survival clinical outcome (Fig 3D P = 0.006). Although the expression of PTPN5 did not correlated with prognosis in ER negative and HER2 subtypes (Fig 3E and 3F), patients with positive clinical outcome in response to tamoxifen therapy in this dataset showed a strong correlation with high levels of PTPN5 (Fig 3G). Finally, using breast cancer classification, we identified good prognostic effect of over expression of PTPN5 in luminal B (P<0.0004) but not in other subtypes of breast cancer patients (Fig 4).

### *Ptpn5* is a novel ERα target gene that is selectively upregulated by tamoxifen and suppresses epidermal growth factor signaling in human breast cancer cells

**Fig 5:**
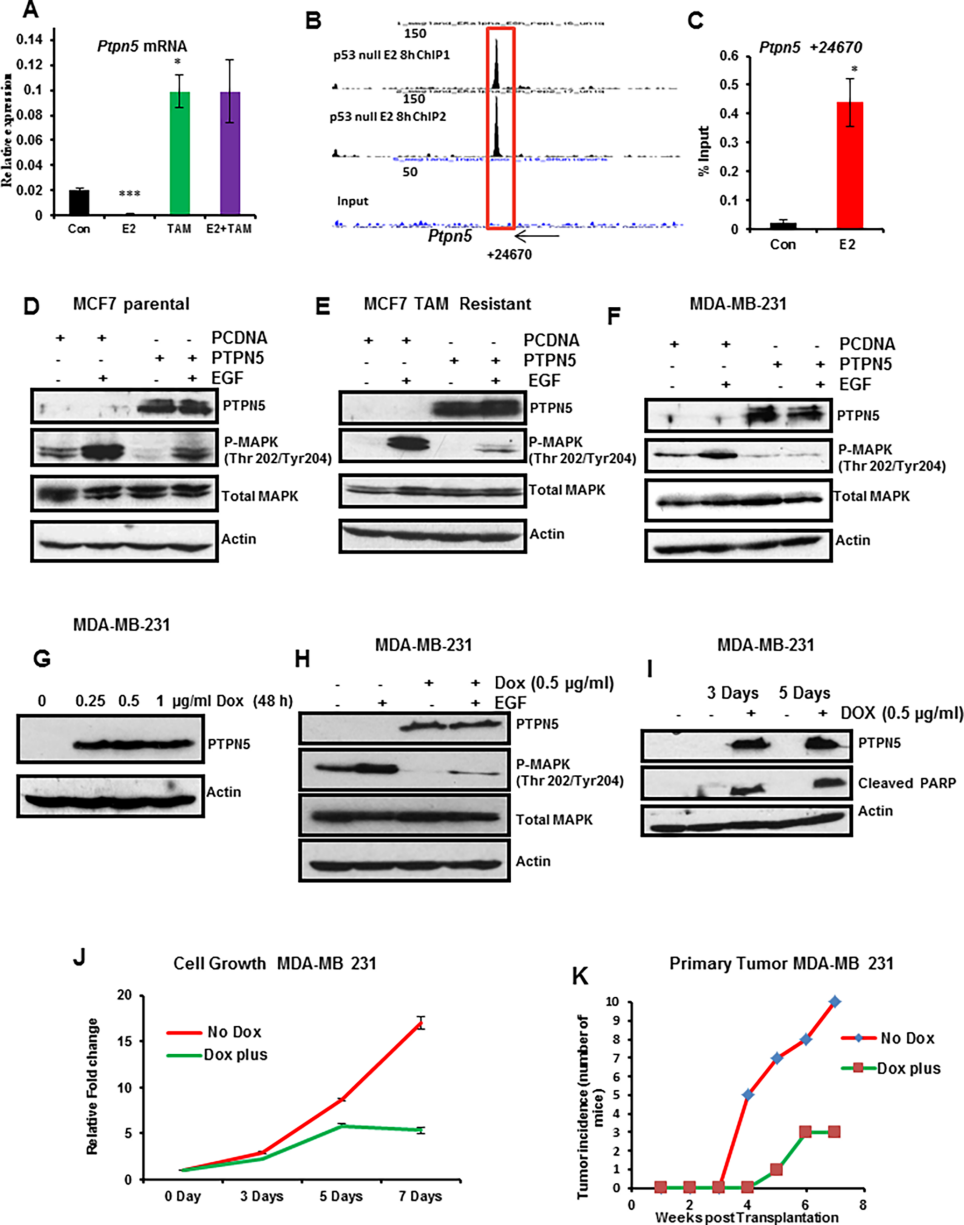
PTPN5 inhibits MAPK signaling in breast cancer cells and suppresses cell growth and tumor growth in TNBC cells. (A) Effect of acute exposure to estradiol, tamoxifen and a combination of both on *Ptpn5* mRNA level in WT mammary gland. Mice (WT 10 week old) were ovariectomized and then rested for 10 days, treated with vehicle or E_2_ (100ng) or TAM (1μg) or combination of both for 24 h. Mammary gland total RNA was extracted and qPCR was performed for *Ptpn5*. (B) UCSC Genome Browser screen shots representing ERα binding in relation to the TSS of *Ptpn5*. Peak locations relative to the TSS are listed below the screen shot and peak values are listed in each peak. Box represents peak that was validated by ChIP-qPCR in WT mammary gland. (C) Mice (WT 10 week old) were OVX and then rested for 10 days, treated with vehicle or E_2_ (100ng) for 8 h. Validation of distal ERα binding using ERα ChIP followed by qPCR for *Ptpn5*. PTPN5 prevents EGF-induced MAPK signaling. (D-F) MCF7 parental, MCF7 tamoxifen resistant and MDA-MB-231 cells were transfected with empty vector (control) or vector containing PTPN5-cDNA for 24h, starved of growth factors and then treated with or without EGF for 15 min. Proteins (50 μg) were separated by SDS-PAGE and then immunoblotted with PTPN5, phosphorylated MAPK (Thr 202/Tyr204), total MAPK and actin antibodies. Protein loading was monitored by stripping and reprobing the same blot with antibodies for MAPK and actin. (G) MDA-MB-231- cells were engineered with an *i*nducible *P*TPN5-cDNA that expresses PTPN5 upon addition of Dox at different concentrations for 48h. PTPN5 and actin (loading control) protein levels were determined by Western blot. (H) Restoring PTPN5 expression suppresses EGF-induced MAPK signaling. MDA-MB-231 cells were transduced with *i*nducible *P*TPN5 and cultured with or without Dox for 48h, and then treated in the presence and absence of EGF for 15 min. The cell lysates were examined for PTPN5, phosphorylated MAPK (Thr 202/Tyr204), total MAPK and actin expression by immunoblot analysis. (I) Restoring PTPN5 expression induces apoptosis. MDA-MB-231 cells were transduced with *i*nducible *P*TPN5 and cultured with or without Dox for 3 and 5 days. Whole cell lysates were analyzed by Western blot for PTPN5, cleaved PARP and actin (loading control) protein levels. (J) Restoring PTPN5 inhibits cell growth. MDA-MB-231 cells were transduced with *i*nducible *P*TPN5 and cultured with or without Dox for 0, 3, 5 and 7 days. Total cell number was counted by Coulter counter. (K) Restoring PTPN5 expression suppresses primary tumor growth in MDA-MB 231 cells. Cells from (G) were transplanted into the mouse mammary gland and monitored for primary tumor growth in the presence or absence of Dox (n = 10 for each group). The blots represent one of three separate experiments. Results are means ± SEM of three independent experimental replicates.

PTPN5 has been implicated in negative regulation of a broad spectrum of receptor tyrosine kinase (RTK) signaling pathways as well as phosphatase-dependent activation of the cell death mediator BAK [42]. We further demonstrated that *Ptpn5* is a direct target of ERα whose expression is acutely repressed by E_2_ and strongly upregulated by tamoxifen in the WT mammary gland (Fig 5A) indicating that *Ptpn5* is induced by TAM not only in p53 null mammary gland but also in WT mammary gland. To determine whether PTPN5 is a direct target of ERα, we interrogated genome-wide chromatin binding of ERα obtained by ChIP-seq analysis of p53 null mammary glands (unpublished data). We identified an ERα binding site located in the distal downstream region of the Ptpn5 gene (Fig 5B). To validate this ERα binding site, mammary gland chromatin was isolated from ovariectomized WT mice treated with vehicle or E_2_ for 8h and then subjected to ERα ChIP followed by qPCR. As shown in Fig 5C, ERα was highly enriched in this region in response to E_2_ treatment, confirming direct ERα recruitment to *Ptpn5*. Thus, *Ptpn5* is a direct target of ERα in both WT and p53 null mammary glands.

To determine whether PTPN5 controls EGF-induced MAPK signaling in human breast cancer cells, we assessed the phosphorylation status of MAPK in various breast cancer cells by forced expression of PTPN5. In these experiments, we used tamoxifen sensitive (MCF7 parental), tamoxifen resistant MCF7 and MDA-MB-231 breast cancer cells. These cells were transfected with empty vector (pcDNA) or pcDNA containing PTPN5 for 24 h followed by EGF treatment for 15 min. Cell lysates were examined for PTPN5 and phospho MAPK by Western blot analysis. As shown in Fig 5D–5F, the level of PTPN5 expression was dramatically increased in cells transfected with PTPN5 vector compared with cells transfected with empty vector. Furthermore, ectopic expression of PTPN5 blocked the phosphorylation of MAPK in response to EGF treatment. Taken together, these results indicate that restoration of PTPN5 function inhibits MAPK signaling in response to EGF treatment in tamoxifen sensitive, resistant and triple-negative breast cancer (TNBC) cells.

### PTPN5 inhibits growth of p53 mutant MDA-MB-231 cells and suppresses tumor growth in a xenograft mouse model

We next examined the functional consequences of PTPN5 activation in the p53 mutant MDA-MB-231 cell line. MDA-MB-231 cells were transduced with inducible lentiviruses expressing PTPN5 and then cells were treated with different concentrations of Dox (0, 0.25, 0.5 and 1μg/ml) for 48 h. We confirmed that Dox-mediated induction of PTPN5 in transduced cells inhibited EGF-dependent activation of MAPK signaling (Fig 5G and 5H). We further examined whether restoration of PTPN5 activates apoptosis in MDA-MB-231 cells. As shown in Fig 5I, PTPN5 was expressed by addition of Dox and was accompanied with increased levels of cleaved PARP, suggesting that restoration of PTPN5 induces apoptosis in MDA-MB-231 cells. Next, we investigated whether restoration of PTPN5 inhibits growth of breast cancer cells. Lentiviral transduced MDA-MB-231 cells were treated with or without Dox (0.5 ug/ml) for 0, 3, 5 and 7 days. As shown in Fig 5J, expression of PTPN5 significantly reduced cell growth indicating that TNBC cells were sensitive to PTPN5 function *in vitro*. Finally, we tested whether PTPN5 restoration suppressed tumor growth *in vivo*. To assess their tumorigenic potential, lentiviral transduced MDA-MD-231 cells were transplanted orthotopically into the #4 mammary gland in mice. The mice were divided into two groups (N = 10 per group) and Dox (2mg/ml) was added to the drinking water of one group. As shown in Fig 5K, all 10 control mice rapidly developed palpable tumors within 7 weeks of transplantation. In contrast, the incidence of tumor development in Dox treated mice was substantially decreased with only 3/10 mice developing tumors over the course of the experiment. Taken together, these data indicate that restoring PTPN5 function inhibits BC cell growth *in vitro* and strongly suppresses tumor growth *in vivo*.

## Discussion

The majority of breast tumors (>70%) rely on ERα signaling for growth. Blockade of estrogen activated ERα function using SERMS such as tamoxifen, is effective in the prevention and treatment of ERα positive breast cancers [43–46]. Nevertheless, resistance to antiestrogen therapy occurs in over half of patients resulting in poor outcomes. Recent studies, relying primarily on human cell culture models of tamoxifen resistant breast cancer cells, have revealed that the acquisition of antiestrogen resistance is associated with emergence of distinct ERα responsive transcriptional programs in breast cancer cells that are associated with genome-wide epigenetic reprogramming of chromatin, including the estrogen responsive enhancer landscape in resistant cells [47–49]. These findings have also recently been extended to primary human breast tumors where differential enhancers binding of ERα and changes in transcriptional output have been associated with distinct clinical outcomes with respect to endocrine therapy [50, 51]. While it is becoming clear from these studies that distinct ERα driven epigenetic signaling programs distinguish antiestrogen outcome in the therapeutic setting, relatively little is known regarding the antiestrogen-dependent mechanisms that underlie prevention against tumor development.

The murine p53 null mammary transplant approach provides an excellent mouse model to address the molecular and epigenetic mechanisms underlying tamoxifen-dependent breast cancer prevention. The p53 null mammary epithelium displays normal postnatal hormone-dependent morphogenesis with few genetic changes relative to WT epithelium [26, 52, 53]. However, in contrast to WT mammary glands, the p53 null mammary epithelium gives rise to hormone-dependent ductal hyperplasia that progress to a heterogeneous group of ERα positive and ERα negative breast cancers with molecular signatures that reflect the luminal, basal and claudin–low subtypes observed in humans [25, 54]. Most importantly in the context of this study, the antiestrogen, tamoxifen is highly protective against development of both ERα positive and ERα negative tumors in this model [28].

In the present study, we used a genome-wide transcription profiling to determine whether global changes in the ERα responsive epigenome contribute to the tumor protective effects observed upon transient exposure of p53 null mammary glands to tamoxifen. We have shown that transient exposure of p53 null mammary glands to tamoxifen leads to a marked decrease in side-branching morphogenesis and epithelial cell proliferation that persist after tamoxifen withdrawal. These morphological and proliferative changes in the mammary epithelium are associated with intrinsic changes in a subset of ERα responsive gene expression signatures.

We observed that transient exposure to tamoxifen leads to persistent changes in the expression of a subset of ERα regulated gene signatures. Among these, we observed persistent changes in estrogen responsive growth factor signaling pathways including downregulation of amphiregulin, an essential ERα regulated mediator of estrogen induced proliferation in the mammary gland [55, 56] and upregulation of growth inhibitory factors including *Ptpn5*. Protein tyrosine phosphatases (PTPs) are a large family of enzymes which generally antagonize the actions of protein tyrosine kinases (PTKs). PTPs are responsible for modulating PTK activity by dephosphorylating various tyrosine residues on intracellular proteins. They also regulate ligand-mediated receptor signaling as well as cell cycle events [57]. Deregulation of PTPs has been implicated in the pathogenesis of various diseases, including breast cancer [58]. A recent study showed that PTPN12 is frequently compromised in a subset of TNBC by deletions, defective sequence variants, or loss of expression [41]. This suggests that HER2/EGFR and other RTK signaling pathways are aberrantly activated in non-HER2 amplified breast cancers [41]. We identified *Ptpn5* as a novel direct target of ERα whose expression is repressed by estrogen and upregulated by tamoxifen in the mammary gland. Our ERα ChIP-seq analysis identified an ERα binding site located in the distal downstream region of the *Ptpn5* gene that may regulate PTPN5 expression via long range chromatin interactions [59].

We found that PTPN5 expression is strongly correlated with improved DMFS in tamoxifen treated breast cancer patients suggesting that PTPN5 may represent a novel prognostic marker of tamoxifen response. Functional validation of PTPN5 in tamoxifen sensitive, tamoxifen resistant and TNBC cells shows that activation of PTPN5 suppressed the EGF-induced MAPK signaling pathway in breast cancer cells. Further, activation of PTPN5 potently inhibits tumor growth of breast cancer growth in a xenograft mouse model of p53 mutant TNBC, suggesting that PTPN5 acts as a tumor suppressor of breast cancer.

In conclusion, our findings reveal that transient exposure to tamoxifen leads to a persistent reduction in mammary gland side branching morphogenesis and MEC proliferation which is associated with intrinsic changes in expression of a subset of estrogen regulated target genes in MECs after tamoxifen withdrawal. *Ptpn5*, a novel upregulated gene in tamoxifen exposed MECs, was identified as a novel potential biomarker of tamoxifen response with tumor suppressor activity.

## Supporting information

S1 Fig Transient exposure to tamoxifen inhibits mammary ductal side-branching in the p53 null mammary gland. (A) Schematic view of the p53 null transplantation into WT mice. At 3 weeks of age, the epithelial ducts (rudiments) of host mice (WT Balb/c) were surgically removed from the inguinal (#4) mammary glands. After 5 weeks, a small mammary fragment (1-mm^2^) from a p53 null Balb/c (8 weeks old) mouse was transplanted into the empty fat pad. At 16 weeks of host age, mice were treated with tamoxifen (5 mg) or sham (control) pellet SC on the back for 90 days. After 4 or 8 weeks of tamoxifen withdrawal, all mice were treated with E_2_ (100ug) for 8h. p53 null transplanted mammary glands were used in this study. (B) Representative whole-mount images of carmine-stained p53 null mammary glands harvested 4 weeks after withdrawal of sham or tamoxifen pellets. Representative glands are shown from two individual mice per treatment.
(PPTX)

S2 Fig Transient exposure to tamoxifen inhibits p53 null MECs proliferation. At 8 weeks after tamoxifen removal, all mice were treated with E_2_ (100ug) for 8 h, #4 mammary glands were harvested, MECs were isolated by collagenase digestion and PCNA, Ki67, cyclin D1 and actin (loading control) expression were analyzed by Western blot. Five mice per pool, tested in duplicate per treatment group.
(PPTX)

S3 Fig Transient exposure to tamoxifen leads to a persistent upregulation of subset of genes in p53 MECs. At 8 weeks after tamoxifen removal, all mice were injected with E_2_ (100ug) for 8 h, #4 mammary glands were harvested, MECs were isolated by collagenase digestion and *Ptpn5*, *Accn1*, *Krt4* and *Fgf12* mRNA levels were analyzed by qPCR. Expression of selected genes was normalized using *Ppid* as the internal control. Five mice per pool, tested in triplicate per treatment group. Results are means ± SEM of three independent experimental replicates. *, *P* < 0.05; ***, *P* < 0.001.
(PPTX)

S4 Fig PTPN5 expression in p53 null mammary gland. A.Representative immunohistochemical staining for PTPN5 on paraffin-embedded p53 null transplanted mammary gland sections from sham and tamoxifen treated mice.
(PPTX)

S1 Table Differentially regulated genes in p53 null MECs. (XLSX)

S2 Table Tamoxifen upregulated genes associated with good prognosis. (XLSX)
