# Supplementary material for: Reprogramming of the estrogen responsive transcriptome contributes to tamoxifen-dependent protection against tumorigenesis in the p53 null mammary epithelial cells
Source: PLoS One. 2018 Mar 28;13(3):e0194913. doi: 10.1371/journal.pone.0194913 (PMC5874056; doi:10.1371/journal.pone.0194913)
Supplement: S1 Fig — (A) Schematic view of the p53 null transplantation into WT mice. At 3 weeks of age, the epithelial ducts (rudiments) of host mice (WT Balb/c) were surgically removed from the inguinal (#4) mammary glands. After 5 weeks, a small mammary fragment (1-mm2) from a p53 null Balb/c (8 weeks old) mouse was transplanted into the empty fat pad. At 16 weeks of host age, mice were treated with tamoxifen (5 mg) or sham (control) pellet SC on the back for 90 days. After 4 or 8 weeks of tamoxifen withdrawal, all mice were treated with E2 (100ug) for 8h. p53 null transplanted mammary glands were used in this study. (B) Representative whole-mount images of carmine-stained p53 null mammary glands harvested 4 weeks after withdrawal of sham or tamoxifen pellets. Representative glands are shown from two individual mice per treatment. (PPTX) [file pone.0194913.s001.pptx]

## Slide 1
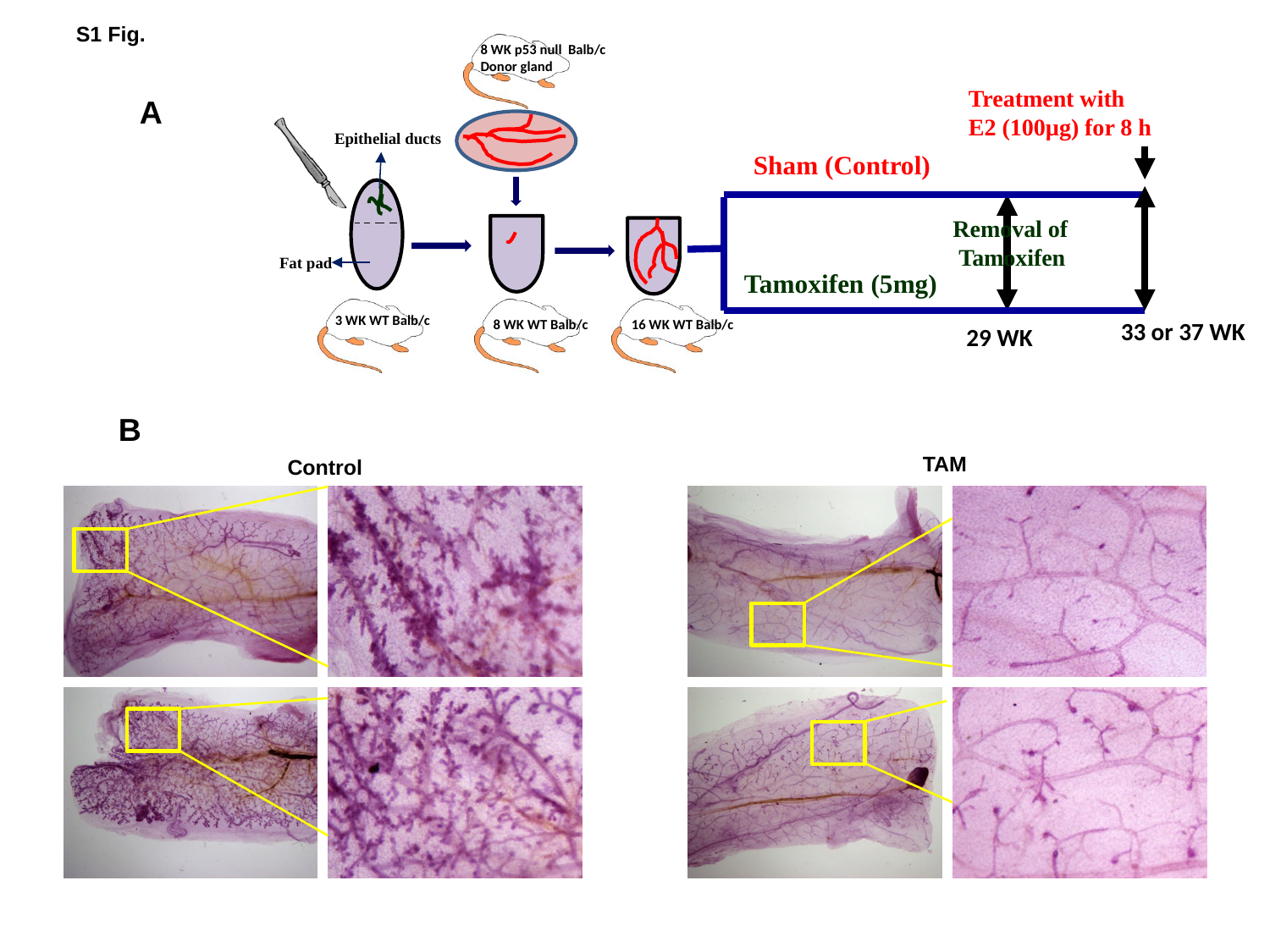

S1 Fig.
8 WK p53 null Balb/c
Donor gland
Treatment with
E2 (100µg) for 8 h
Epithelial ducts
Sham (Control)
Removal of
 Tamoxifen
Fat pad
Tamoxifen (5mg)
 33 or 37 WK
 29 WK
3 WK WT Balb/c
8 WK WT Balb/c
16 WK WT Balb/c
A
B
TAM
Control
