# Supplementary figures and images for: Reprogramming of the estrogen responsive transcriptome contributes to tamoxifen-dependent protection against tumorigenesis in the p53 null mammary epithelial cells
Source: PLoS One. 2018 Mar 28;13(3):e0194913. doi: 10.1371/journal.pone.0194913 (PMC5874056; doi:10.1371/journal.pone.0194913)

## Slide 1
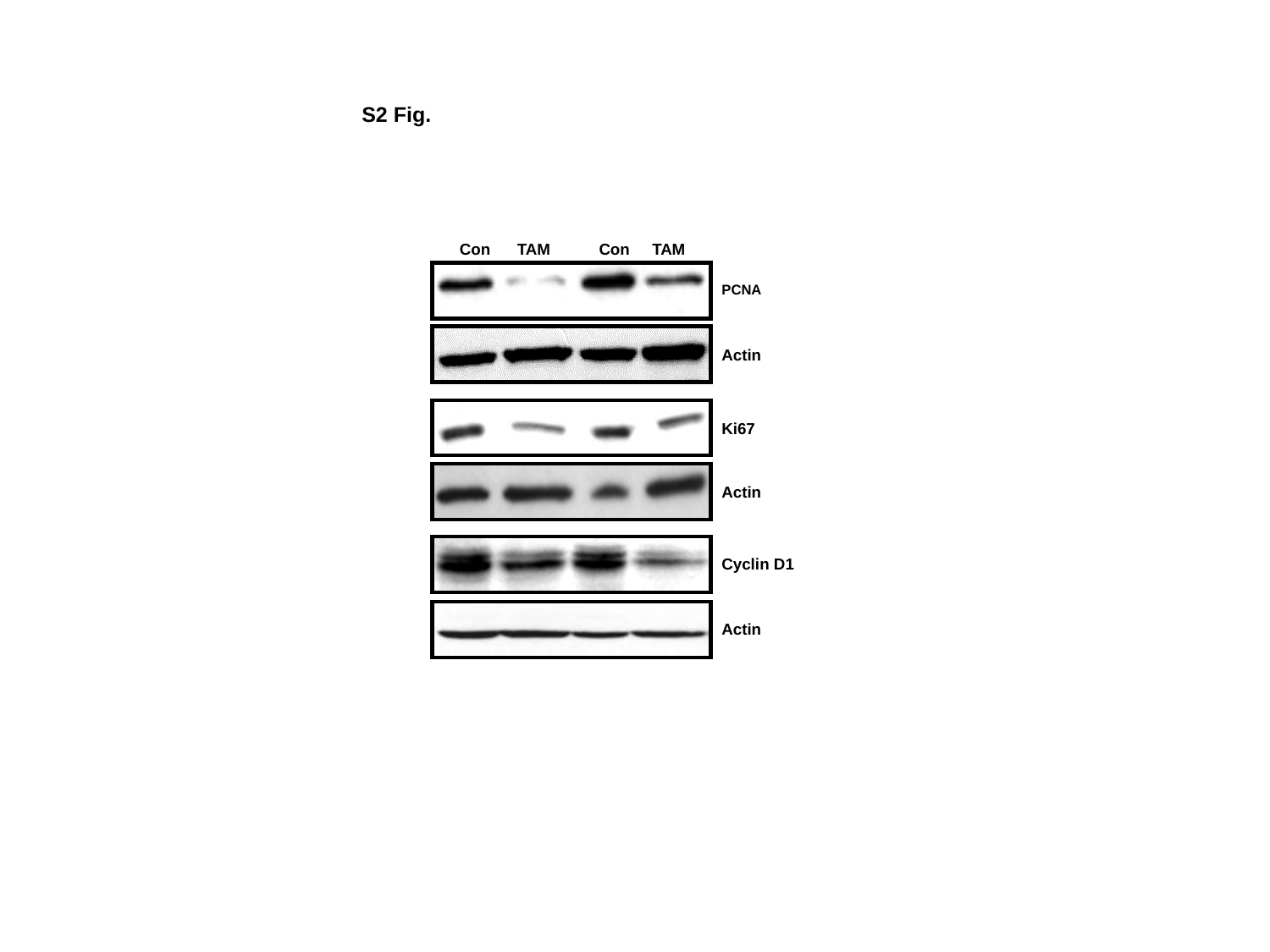

S2 Fig.
 Con TAM Con TAM
PCNA
Actin
Ki67
Actin
Cyclin D1
Actin

Supplement: S2 Fig — At 8 weeks after tamoxifen removal, all mice were treated with E2 (100ug) for 8 h, #4 mammary glands were harvested, MECs were isolated by collagenase digestion and PCNA, Ki67, cyclin D1 and actin (loading control) expression were analyzed by Western blot. Five mice per pool, tested in duplicate per treatment group. (PPTX) [file pone.0194913.s002.pptx]

## Slide 1
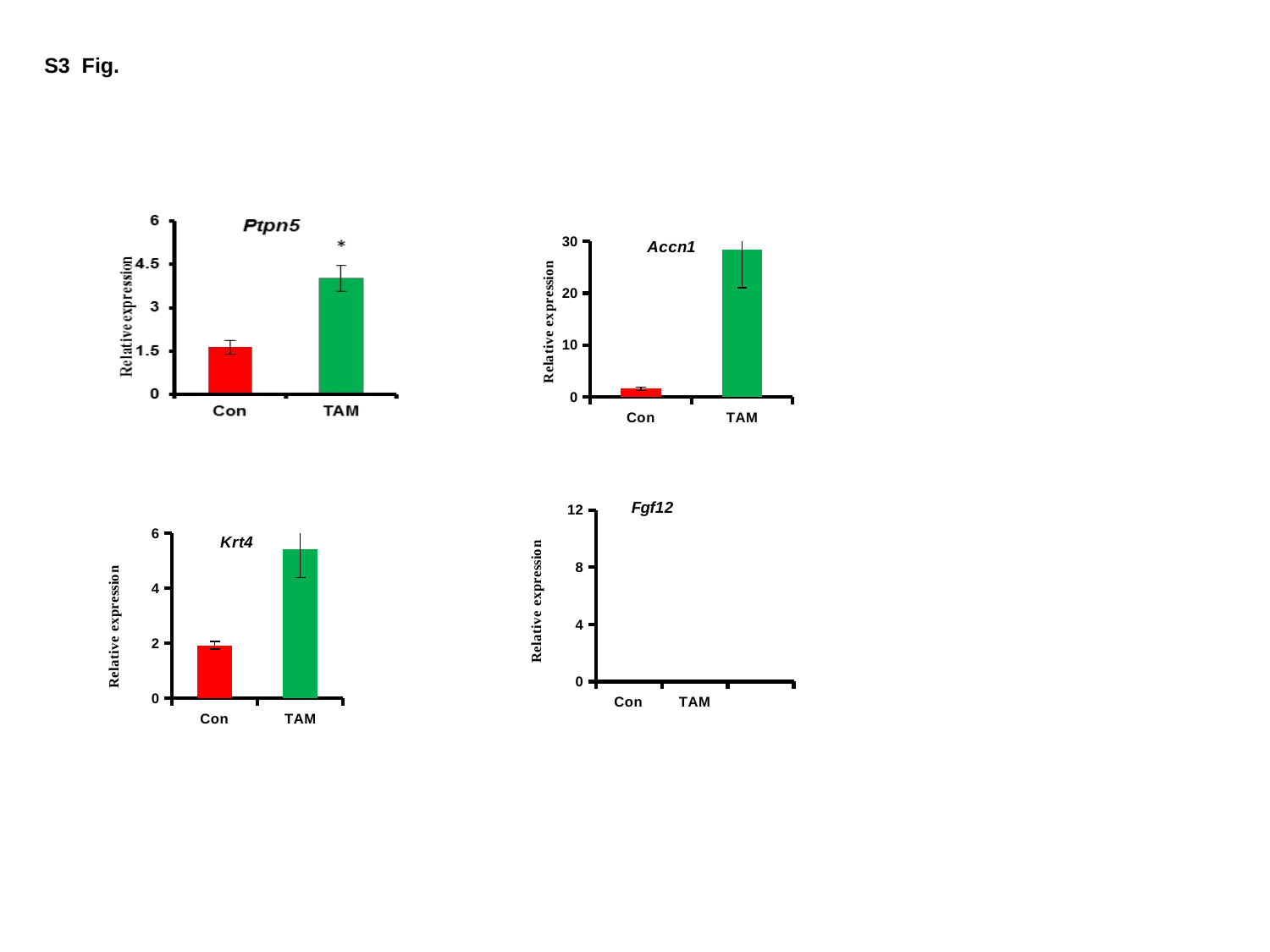

S3 Fig.
### Chart: Accn1
| Category | |
|---|---|
| Con | 1.56 |
| TAM | 28.49 |
### Chart: Fgf12
| Category | |
|---|---|
| Con | 1.62 |
| TAM | 12.74 |
### Chart: Krt4
| Category | |
|---|---|
| Con | 1.9100000000000001 |
| TAM | 5.42 |

Supplement: S3 Fig — At 8 weeks after tamoxifen removal, all mice were injected with E2 (100ug) for 8 h, #4 mammary glands were harvested, MECs were isolated by collagenase digestion and Ptpn5, Accn1, Krt4 and Fgf12 mRNA levels were analyzed by qPCR. Expression of selected genes was normalized using Ppid as the internal control. Five mice per pool, tested in triplicate per treatment group. Results are means ± SEM of three independent experimental replicates. *, P < 0.05; ***, P < 0.001. (PPTX) [file pone.0194913.s003.pptx]

## Slide 1
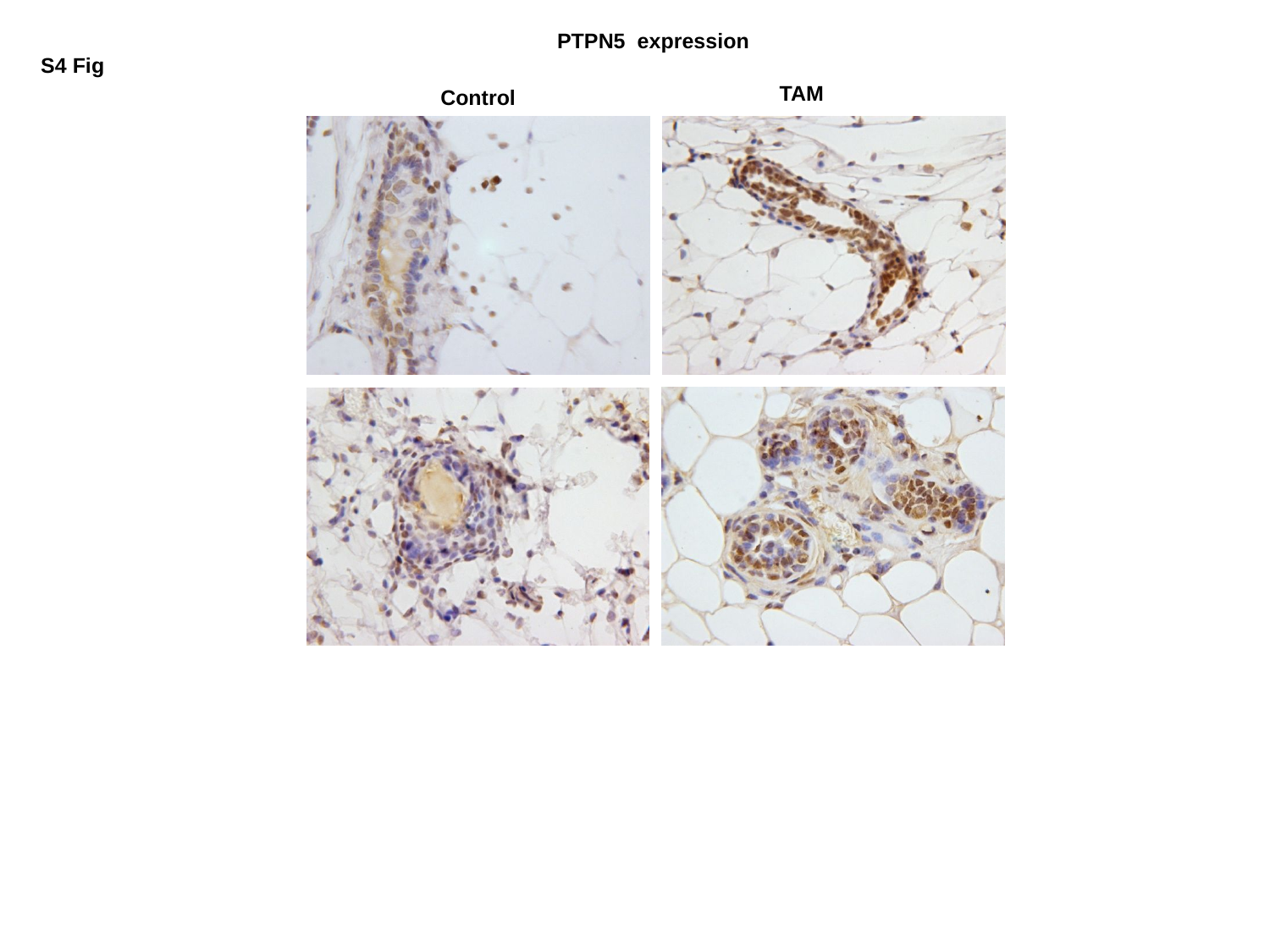

PTPN5 expression
S4 Fig
TAM
Control

Supplement: S4 Fig — A.Representative immunohistochemical staining for PTPN5 on paraffin-embedded p53 null transplanted mammary gland sections from sham and tamoxifen treated mice. (PPTX) [file pone.0194913.s004.pptx]
